# The Principles’ and Practice of Surgery

**Published:** 1886-06

**Authors:** 


**The Principles and Practice of Surgery.** By FRANK H. HAMILTON, M. D., LL.D., late Professor of the Practice of Surgery with Operations, and of Clinical Surgery in Bellevue Hospital Medical College; Consulting Surgeon to Bellevue Hospital; to the Bureau of Surgical and Medical Relief for the Out-door Poor at Bellevue Hospital, etc. Illustrated with 422 engravings on wood. Third edition, revised and corrected. New York: William Wood & Co. 1886.

We have before us the third and revised edition of this work, by one of America's most distinguished authors. He needs no introduction to our readers, and, in all probability, the most of our older physicians have the earlier editions of this work in their library. By comparison, however, it will be found to be, in many respects, too old for practical use, being fourteen years old. The present edition is fully up to date, and is a useful and necessary book to possess. Some parts of the work are regarded as the best on the subject. Again, the position held by Dr. Hamilton is not supported by the majority of surgeons—as in the case of his opposition to antiseptic dressing, etc. He opposes Dr. Otis' method of treating strictures of the urethra. It is a well-known fact that Dr. Otis has wonderful success in the treatment of strictures of the urethra by his method, but that in the hands of others it has not been so successful. Chapter XII, on the nervous system, is entirely new, and excellent. It includes tetanus shock, section of the nerves, stretching of the nerves, etc. The chapters on fractures and dislocations are all that could be desired. On these subjects Dr. Hamilton is justly given the foremost place. The reader gets the impression throughout the book that this is a conservative surgery, and a safe guide for all surgeons and practitioners. Taking it all in all, it is not saying too much if we say it is, perhaps, the best *hand-book* of surgery now published. The engravings and topographical work are all that could be desired.

